# Binocular vision and foraging in ducks, geese and swans (Anatidae)

**DOI:** 10.1098/rspb.2023.1213

**Published:** 2023-09-06

**Authors:** Jennifer C. Cantlay, Graham R. Martin, Stephanie C. McClelland, Simon Potier, Michelle F. O'Brien, Esteban Fernández-Juricic, Alexander L. Bond, Steven J. Portugal

**Affiliations:** ^1^ Department of Biological Sciences, School of Life and Environmental Sciences, Royal Holloway University of London, Egham, Surrey TW20 0EX, UK; ^2^ School of Biosciences, University of Birmingham, Edgbaston, Birmingham B15 2TT, UK; ^3^ Department of Biology, Faculty of Science, Lund University, Sölvegatan 35, 223 62 Lund, Sweden; ^4^ Wildfowl and Wetlands Trust (WWT), Slimbridge, Gloucestershire GL2 7BT, UK; ^5^ Department of Biological Sciences, Purdue University, West Lafayette, IN, USA; ^6^ Bird Group, Department of Life Sciences, The Natural History Museum, Akeman Street, Tring, Hertfordshire HP23 6AP, UK

**Keywords:** Anatidae, binocularity, blind area, ophthalmoscope, visual fields

## Abstract

Wide variation in visual field configuration across avian species is hypothesized to be driven primarily by foraging ecology and predator detection. While some studies of selected taxa have identified relationships between foraging ecology and binocular field characteristics in particular species, few have accounted for the relevance of shared ancestry. We conducted a large-scale, comparative analysis across 39 Anatidae species to investigate the relationship between the foraging ecology traits of diet or behaviour and binocular field parameters, while controlling for phylogeny. We used phylogenetic models to examine correlations between traits and binocular field characteristics, using unidimensional and morphometric approaches. We found that foraging behaviour influenced three parameters of binocular field size: maximum binocular field width, vertical binocular field extent, and angular separation between the eye-bill projection and the direction of maximum binocular field width. Foraging behaviour and body mass each influenced two descriptors of binocular field shape. Phylogenetic relatedness had minimal influence on binocular field size and shape, apart from vertical binocular field extent. Binocular field differences are associated with specific foraging behaviours, as related to the perceptual challenges of obtaining different food items from aquatic and terrestrial environments.

## 1. Introduction

Morphological and phylogenetic diversity within the class Aves [1,2] has provided an ideal framework to investigate form and function associations using a trait-based approach [3–5]. While phylogenetically informed comparative studies have examined evolutionary relationships between the key function of foraging and anatomical traits of skull and bill shape [6,7], its application to sensory organ structures has been largely neglected. Together with communication [8,9], foraging is an important driver for sensory capacities in birds and is often traded off against predator detection [10]. Although several avian species depend on tactile [11,12], olfactory [13,14] and/or auditory cues [15,16] for feeding, many others rely primarily upon vision to acquire their food [17]. Variation within the ocular structures of birds [18–21] highlights the opportunities for large-scale, trait-based comparative analyses of visual anatomy and properties associated with key tasks, such as foraging. Visual fields present a useful trait to study, since differences in visual field characteristics have been described across a wide range of bird species and appear to be related to foraging ecology [17].

The visual field is an important component of every visual system, since its overall shape and extent defines the three-dimensional space around the head from which visual information can be extracted at any instant [10]. Variation in eye size, optical structure and position within the skull determine the way in which visual fields of individual eyes overlap [22,23], thus affecting not only the view around the head but also the shape, size and position of the binocular field. Interspecific differences in the size of specific visual field features (the monocular, binocular, cyclopean fields and blind area; see table 1 for definitions) are associated with specific tasks, primarily foraging, chick provisioning, predator detection and tracking conspecifics [24–27]. Variation in visual field characteristics have also been found in closely related species that occupy specific ecological niches for obtaining food resources, for example, among ibises and spoonbills (Threskiornithidae [28]), ducks, geese and swans (Anatidae [29]), finches (Emberizidae [21]) and birds of prey (Accipitridae and Cathartidae [30]). Within these groups, numerous examples exist which suggest that foraging ecology rather than phylogeny determine visual field parameters [10]. The visual fields of Aegypiinae vultures, for example, are primarily adapted for their obligate carrion-feeding diet, characteristically containing a small binocular region and large blind areas above, below and behind the head [31]. However, active hunting white-headed vultures (*Trigonoceps occipitalis*) have visual fields more similar to that of active-hunting predatory raptors (e.g. accipitrid hawks), rather than their close relatives [31]. More specifically, variation in the visual field topography of Anatidae has been found to occur between species considered to be primarily reliant upon tactile or visual cues. Visually guided foraging ducks, including Eurasian wigeons (*Mareca Penelope*) (selective grazers), blue ducks (*Hymenolaimus malacorhynchos*) and long-tailed ducks (*Clangula hyemalis*) (both dive for prey), have frontally positioned eyes and a wide binocular field with the bill tip located close to the centre of the frontal binocular for the control of bill position to obtain food items ([10] and references therein). Filter-feeding mallards (*Anas platyrhynchos*), northern shovelers (*Spatula clypeata*) and pink-eared ducks (*Malacorhynchus membranaceus*) have dorsally positioned eyes and a narrow binocular field with an extensive vertical length to provide comprehensive visual coverage of the celestial hemisphere ([10] and references therein). This literature supports the concept that interspecific binocular field variation is primarily associated with the perceptual challenges of foraging, rather than phylogenetic relatedness.

**Table 1.** Full definitions of terms relating to visual field parameters.

term	definition
monocular field	the visual field of a single eye
binocular field	the area where monocular fields overlap
cyclopean field	the total visual field produced by the combination of both monocular fields
maximum binocular field width	the maximum binocular field width measured within the binocular region
angular separation	the location of the bill tip projection within, or even outside of the binocular field, with respect to the position of maximum horizontal field width
vertical binocular field extent	the degree to which binocularity extends from beneath the bill to above the head

Binocular field topography varies among species associated with the perceptual challenge of controlling bill (or talons) position during specific foraging activities [23] and/or chick feeding [26]. Binocularity enables accurate control of the direction of travel of the bill (or feet), and their time to contact with a target [25]. This is based on the bird's detection of symmetrical optic flow-fields generated by movement of the head towards a target [10]. Consequently, binocular field topography represents an important trait for further understanding the evolution of avian vision. Binocularity is considered the most pivotal component of the avian visual field. This is owing to the two vital functions binocularity provides; (i) tasks associated with locomotion and (ii) the accurate placement of the bill with respect to the target [10]. Combined, these two components provide the capacity to move, forage, construct nests and feed young.

A phylogenetically broad assessment of visual field characteristics and ecological traits, such as foraging, has not been conducted prior to this study. To date, analyses of particular visual field characteristics have involved targeted comparisons among species selected for their ecology or phylogeny [23,32–34]. These comparisons have examined single parameters, such as binocular field width in the plane of the bill, maximum binocular field width, binocular field vertical extent, blind area width (directly above and behind the head), lateral field width and cyclopean field width [21,24,35,36]. More recently, a multidimensional approach to binocular field analysis among birds of prey (Accipitriformes species [30]) provided new insights into binocular field shape associated with specific foraging techniques. This morphometric analysis of binocular field shape revealed additional information that was not evident when using unidimensional (single parameter) comparisons of visual fields. Few visual field comparative analyses have included a phylogenetic component [21,30,37], which limits our understanding of evolutionary drivers on visual field function.

The ecomorphological diversity within the ducks, geese and swans (Anatidae [38]) provides an appropriate model taxon for the trait-based comparative analysis of binocular fields. Since they have precocial chicks [39], binocular vision is not required for chick provisioning as occurs in other taxa [26]. This allows us to focus on the association between binocular fields and foraging traits. The wide range of foraging techniques, diets [38,39] and anatomical morphologies, e.g. skull shape [40], bill structure [12,41] and gastrointestinal tract [41,42], enables the Anatidae to exploit a wide variety of foraging niches found in marine, freshwater and terrestrial habitats [43]. Variation in binocular field topography (related to bill tip position, binocular field width and vertical extent) of the Anatidae has been described in species considered to either be primarily reliant upon tactile or visual cues [29,44–46]. This suggests that interspecific binocular field variation may be associated with the perceptual challenges of foraging, rather than phylogenetic relatedness [10,25]. We aim to provide, to our knowledge, the first phylogenetically informed comparative study of Anatidae species to test this hypothesis using both unidimensional and multidimensional approaches.

We hypothesized that both diet and foraging behaviour would have a more predominant role in the evolution of binocular field dimensions for Anatidae species than phylogenetic relatedness. Our analyses firstly examines three descriptors of binocular field size (maximum binocular field width, vertical binocular field extent and angular separation between the eye-bill projection and the direction of maximum binocular field width), and secondly explores the descriptor of binocular field shape.

## 2. Material and methods

### (a) Species and study locations

Research was conducted at two locations: U.S. Geological Survey Patuxent Wildlife Research Center, Maryland, USA (October 2018) and the Wildfowl and Wetlands Trust, Slimbridge, Gloucestershire, UK (July 2016, September 2018, January, February and December 2019). Species and the number of individuals studied at these locations are listed in the electronic supplementary material, table S1. Visual field measurements were obtained from 39 species (one swan, seven geese and 31 ducks), using new data collected from 33 species and previously published data from six species (electronic supplementary material, table S1). The rationale and relevance of the binocularity are further discussed in the electronic supplementary material, S1.

### (b) Binocular field data

The ophthalmoscopic reflex technique was used to measure the visual field characteristics of individuals for each species. This followed the standardized and validated method as described in detail in previous studies [28,34,44] (see the electronic supplementary material, methods S2). Individual birds were measured close to their enclosures and immediately returned there following the completion of data collection.

**Figure 1.**
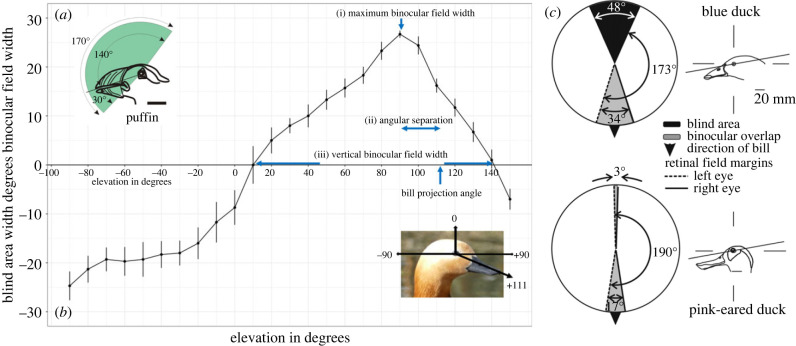
(*a*) Vertical sections through the binocular fields of an Atlantic puffin, in the median sagittal plane defined by the vertically orientated equator of the diagrams (taken from [23]). The line drawings of the heads of the bird show them in the approximate orientations adopted when the visual fields were measured. (*b*) The mean ± s.e. angular separation of the retinal field margins as a function of elevation in the median sagittal plane in ruddy shelducks (*n* = 3). Positive values indicate overlap of the field margins (binocular vision) and negative values indicate the blind area width. The coordinate system is such that the horizontal plane is defined by the elevations −90° (behind the head), +90° (in front of the head), and 0° (directly above the head), shown in the photograph. The projection of the eye-bill tip projection is 111°. The graph illustrates the three visual field parameters measured for this species: (i) the width of maximum binocular field overlap = 27°, (ii) the angular separation between the eye-bill projection and elevation of the maximum binocular field width (111 − 90° = 21°), and (iii) the vertical extent of the binocular field = 131°. (*c*) Horizontal sections through the visual fields of blue ducks and pink-eared ducks in a plane containing the frontal binocular field at its maximum width (taken from [44]). This plane is indicated by the line through the eye in each of the drawings of the birds to the right of each visual field diagram.

Interspecific variation in binocular field topography was assessed using both unidimensional and multidimensional approaches. Unidimensional analysis was based on three binocular field parameters that were selected as response variables [24,30,32]: the width of maximum binocular field overlap; the angular separation between the eye-bill projection and the direction of maximum binocular field width; and the vertical extent of the binocular field (figure 1). Angular separation defines the location of the bill tip projection within, or even outside of the binocular field, with respect to the position of maximum horizontal field width (figure 1). Negative values represent the number of degrees the bill tip projection is above the maximum binocular field width, while positive values represent the number of degrees the bill tip projects below the maximum binocular field width, when the bird's head is held in a natural resting position. For each species, mean values of these three visual field parameters were used. The multidimensional, morphometric approach provided a comparison of mean binocular field shapes for each species (see below). This used data from each 10° elevation in the median sagittal plane of the head for each species (figure 1).

### (c) Life-history data

Foraging behaviour information was gathered from primary literature [38,39,47]. The most common foraging methods were: grazing (seizing of terrestrial vegetation), dabbling (seizing of items at a water surface), filter feeding (extraction of small items from the water column or from within water-filled substrates) and diving (either pursuit diving for capture of mobile prey at depth in the water column, or shallow diving to feed on vegetation or prey attached to or within the substrate) [39,48]. Subsequently, the primary foraging behaviour was determined for each species and classified into one of four categories: ‘dabble filter’, ‘dive open’, ‘dive substrate’ and ‘graze’. Dabble filter (*n* = 15) includes both dabbling (feeding on the surface of the water or tipping headfirst into the water to reach submerged food) and filter feeding (using lamellae in the bill to strain water for food). Dive open (*n* = 3) refers to birds diving underwater to capture prey from the water column, while dive substrate (*n* = 12) refers to bird diving underwater to obtain food from the substrate. Graze (*n* = 9) refers to the grazing of vegetation in the terrestrial environment. These indicate whether foraging primarily occurs in terrestrial (grazing) or aquatic environments (dabbling and filtering, or diving). Dietary categories were classified according to those described in an existing dataset based upon the primary dietary component [49], which provided three categories: ‘plant seed’ (*n* = 24), ‘invertebrate’ (*n* = 11) and ‘omnivore’ (*n* = 4). Body mass of adult birds was taken as a mean for both sexes [50].

### (d) Phylogenetic methods and analyses

#### (i) Binocular field parameters

Statistical analysis and figure illustrations were conducted in R v. 3.5.3 (R Core Team 2019) using the RStudio environment (R Studio Team 2019). For the analysis, the explanatory variables considered were primary diet, foraging behaviour and body mass, while the response variables were the binocular field parameters of maximum binocular width, angular separation and vertical binocular extent (table 1). We modelled visual field parameters to consider their shared phylogeny, since species are not statistically independent [51,52]. We used the database www.birdtree.org [53,54] for phylogenetic tree construction. Phylogenetic generalized least-squares (PGLS) models were fitted for each binocular field parameter using the function ‘pgls’ from the *caper* package [55]. We fitted PGLS models to test for an association between the three life-history traits (primary diet, foraging behaviour and log_10_ body mass) and the three binocular field response variables (see above). A null model (containing no predictors) was created for each visual field variable to measure the phylogenetic autocorrelation of each response variable alone. Eight PGLS model combinations were tested for each visual field parameter (null model, full model, three models each containing a single life-history trait and three models each containing two traits). PGLS model fitting used the maximum-likelihood estimate of *λ*. Full methodological details pertaining to collinearity, data skewness, model selection and model averaging can be found in the electronic supplementary material, methods S3. Owing to dive open being a small category (*n* = 3), we re-ran the entire analysis (as above) without the dive open category included, to determine the possible impact that this category was having on the results.

#### (ii) Binocular field shape

We used the morphometric approach for the comparative analysis of binocular field shape across species. Binocular field shape provides the geometric morphometric representation of the binocular field in two dimensions [30]. We followed the method previously described for visual fields of Accipitriformes [30], which relied upon outline analysis to translate shapes into quantitative variables in their application to a common multivariate framework for comparative data analyses [56]. Full methodological details shape analyses can be found in the electronic supplementary material, methods S3. Briefly, an elliptic Fourier transform (EFT) was calculated on the *x* and *y* coordinates of the binocular field outline shapes for each species [57]. This process transformed these coordinates into two harmonic sums of trigonometric functions to provide the best approximation of the binocular shape outline [30], following the EFT principle described using the *Momocs* package [56]. We conducted a principal component analysis (PCA) on the matrix of Fourier coefficients to obtain principal component (PC) factors for binocular field shape [30] within the *Momocs* package [56]. Based on eigen factors, the scores of selected axes (PC1 and PC2), were then used in PGLS model analyses for the examination of binocular field shape variation as a function of the three life-history traits (primary diet, foraging behaviour and log_10_ body mass), which followed the statistical method previously described for single binocular field parameters. In total, eight PGLS model combinations were tested for each PC factor (null model, full model, three models each containing a single trait) and three models each containing two and a model selection [58]. For PC1, subsequent conditional model averaging was conducted with pairwise comparisons across diet and foraging categories. Since there was a single top-ranked model for PC2, model averaging was not necessary. All data are available as the electronic supplementary material, and all full statistical outputs from each model can be found in the electronic supplementary material, tables S3–S7.

## 3. Results

### (a) Single binocular field parameters

#### (i) Phylogenetic signal

**Figure 2.**
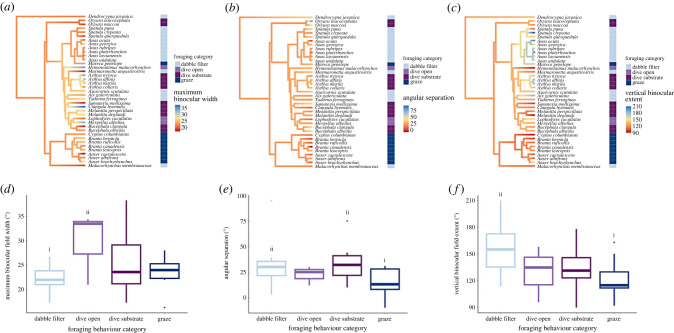
Phylogeny of Anatidae species (top). Branch colours indicate reconstructed ancestral states for binocular field traits (*a*) maximum binocular field width, (*b*) angular separation and (*c*) vertical binocular field extent, with colours at the tree tips representing the current states of these traits. The predictor trait of foraging behaviour is categorized by coloured bars adjacent to the tree. All colour coding is provided in the legend. For the sampled Anatidae species (*n* = 39) (bottom), box plots showing mean (*d*) maximum binocular field width, (*e*) angular separation and (*f*) vertical binocular field extent, across the four primary foraging categories. The letters i and ii reflect significant differences (*p* < 0.05) between categories. An alternate version of (*d–f*) can be found as the electronic supplementary material, figure S1.

Pagel's *λ* was intermediate for vertical binocular extent (*λ* = 0.61), suggesting that this parameter tended to have correlated values in close relatives, although not as correlated as expected under a Brownian motion model of evolutionary change. On the other hand, phylogenetic signal was low for angular separation (*λ* = 0.21) and maximum binocular width (*λ* < 0.001), indicating that these did not tend to be more similar between closely related species, thus suggesting these traits are driven more by life-history traits (see figure 2 for phylogenetic trees).

#### (ii) Maximum binocular field width

The maximum binocular field width ranged from 16° in snow geese *Anser caerulescens* to 38° in long-tailed ducks *C. hyemalis*. Three competitive models (Akaike information criterion (ΔAICc) < 2) were identified from model selection (electronic supplementary material, table S3). Subsequent conditional model averaging showed that foraging behaviour was an important predictor, since species using dabbling/filtering techniques had significantly smaller maximum binocular field widths than species diving in open water (*β* = −7.35*,* s.e. = 3.18, *z* = 2.31, *p* = 0.02). However, there were no significant differences in maximum binocular field width between all other foraging categories (figure 2): dabble/filter feeders and substrate divers (*β* = −2.95, s.e. = 1.96, *z* = 1.50, *p* = 0.13); open water divers and substrate divers (*β* = 4.41, s.e. = 3.26, *z* = 1.35, *p* = 0.18), and grazers and substrate divers (*β* = −2.14, s.e. = 2.31, *z* = 0.93, *p* = 0.36). Variation in maximum binocular field width among foraging categories is illustrated (figure 2*d*). Neither log_10_ body mass (*β* = 0.31, s.e. = 3.16, *z* = 0.10, *p* = 0.92) nor primary diet predicted maximum binocular field widths, as no significant differences were found between dietary categories: invertebrate feeders and plant/seed feeders (*β* = 0.88, s.e. = 2.32, *z* = 0.38, *p* = 0.79); omnivores and plant/seed feeders (*β* = −3.67, s.e. = 2.88, *z* = 1.28, *p* = 0.20); invertebrate feeders and omnivores (*β* = 4.55, s.e. = 3.06, *z* = 1.50, *p* = 0.14).

#### (iii) Angular separation

The angular separation between the eye-bill projection and the direction of maximum binocular field width ranged from −10° (i.e. the bill tip being 10° above the maximum binocular field width elevation) in Canada geese *Branta canadensis* to 95° (i.e. the bill tip being 95° below the maximum binocular field width elevation) in mallards *Anas platyrhynchus*. Four competitive models (ΔAICc < 2) were identified from model selection (electronic supplementary material, table S4). Subsequent conditional model averaging identified that foraging behaviour was a key predictor, as grazing species had significantly smaller angular separation than species using dabbling/filtering techniques (*β* = −15.93, s.e. = 7.77, *z* = 2.05, *p* = 0.04) and those diving in substrate (*β* = −19.68, s.e. = 8.19, *z* = 2.40, *p* = 0.02). There were no significant differences in angular separation between the other foraging categories: dabble/filter feeders and substrate divers (*β* = −3.75, s.e. = 7.12, *z* = 0.53, *p* = 0.60); open water divers and substrate divers (*β* = −12.79, s.e. = 11.34, *z* = 1.13, *p* = 0.26). Variation in angular separation among foraging categories is illustrated (figure 2*e*). Neither log_10_ body mass (*β* = −14.04, s.e. = 11.31, *z* = 1.24, *p* = 0.21) nor primary diet predicted angular separation, since no significant differences were found between dietary categories: invertebrate feeders and plant/seed feeders (*β* = −0.57, s.e. = 7.72, *z* = 0.07, *p* = 0.94), omnivores and plant/seed feeders (*β* = 14.70, s.e. = 10.04, *z* = 1.46, *p* = 0.14) and invertebrate feeders and omnivores (*β* = −15.27, s.e. = 11.13, *z* = 1.37, *p* = 0.17).

#### (iv) Vertical binocular field extent

The vertical binocular field extent ranged from 90° in white-winged scoters *Melanitta deglandi* to 210° in northern shovelers *S. clypeata* and mallards. Three competitive models (ΔAICc < 2) were identified from model selection (electronic supplementary material, table S5). Conditional model averaging showed that dabbling/filter-feeding species had significantly greater vertical binocular extents than grazing species (*β* = 32.93, s.e. = 11.86, *z* = 2.78, *p* = 0.01). There were no significant differences in vertical binocular field extent between the other foraging categories: dabble/filter feeders and substrate divers (*β* = 21.02, s.e. = 10.89, *z* = 1.93, *p* = 0.05); open water divers and substrate divers (*β* = −5.25, s.e. = 18.16, *z* = 0.29, *p* = 0.77); grazers and substrate divers (*β* = −11.92, s.e. = 12.40, *z* = 0.96, *p* = 0.34). Variation in angular separation among foraging categories is illustrated (figure 2*f*). Neither log_10_ body mass (*β* = −14.54, s.e. = 17.29, *z* = 0.84, *p* = 0.40) nor primary diet predicted vertical binocular field extents, as no significant differences were found between dietary categories: invertebrate feeders and plant/seed feeders (*β* = −10.35, s.e. = 10.81, *z* = 0.96, *p* = 0.34); omnivores and plant/seed feeders (*β* = 17.39, s.e. = 14.76, *z* = 1.18, *p* = 0.24), and invertebrate feeders and omnivores (*β* = −27.74, s.e. = 15.87, *z* = 1.75, *p* = 0.08).

#### (v) Binocular field parameters

The reconstructed ancestral states of the binocular field parameters associated with foraging behaviour for the phylogeny of Anatidae are illustrated (figure 2). While the maximum binocular field width and angular separation appeared relatively conserved for species that dabble/filter feed or graze, the vertical binocular extent showed greater variation for species within these foraging groups. Those species diving to forage in substrate showed great variation in maximum binocular field width and vertical binocular extent, yet their angular separation is more conserved. Overall, variation in foraging behaviour categories determined binocular field size across species based on these three binocular field parameters, with relatively little correlation in the residual error of the model (for maximum binocular field width and angular separation) linked to phylogenetic relationships.

### (b) Binocular field shape

**Figure 3.**
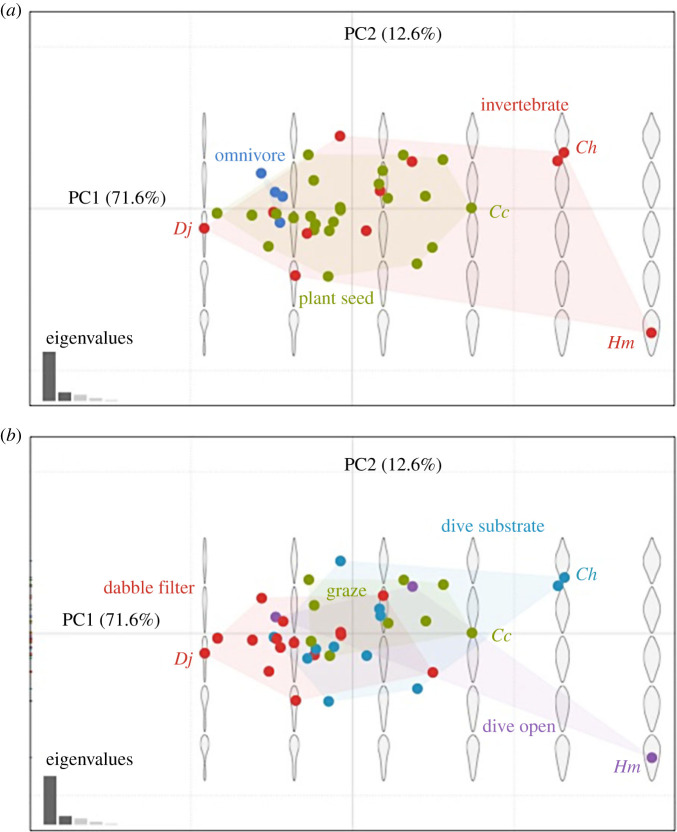
Results of principal component (PC) analysis showing binocular field shapes across 39 Anatidae species with respect to (*a*) primary diet, and (*b*) foraging behaviour categories. Some species with extreme examples of binocular field shape within each diet and foraging category are labelled: *Dendrocygna javanica* (*Dj*), *Cygnus columbianus* (*Cc*), *Clangula hyemalis* (*Ch*) and *Hymenolaimus malacorhynchos* (*Hm)*.

The first two PCs accounted for 84.2% of the total variance (71.6% for PC1, eigenvalue less than 0.0001; 12.6% for PC2, eigenvalue less than 0.0001; figure 3). PCA for binocular field shape showed that positive PC1 scores represent a wider binocular field at and around the horizontal plane with a larger shape at the lower edge (electronic supplementary material, figure S2), and positive PC2 scores represent a narrower shape at the upper edge of the binocular field (electronic supplementary material, figure S2). For both PC1 and PC2, negative scores produced a binocular field with an inverted teardrop shape, while positive scores appeared more elliptical in shape (electronic supplementary material, figure S2).

#### (i) Phylogenetic generalized least-squares analyses

The phylogenetic signal was low for PC1 (*λ* < 0.0001) and PC2 (*λ* < 0.0001), indicating that species values for PC factors are not more similar between closely related species than among distantly related taxa in Anatidae.

For PC1, three competitive models (ΔAICc < 2) were identified from model selection (electronic supplementary material, table S6). Subsequent conditional model averaging identified that foraging behaviour was a key predictor. For species diving in open water and diving to forage in substrate, both had significantly greater PC1 scores than species using dabbling/filtering techniques (dive open *β* = 0.12, s.e. = 0.04, *z* = 2.76, *p* = 0.01; dive substrate *β* = 0.06, s.e. = 0.03, *z* = 2.23, *p* = 0.03). There were no significant differences in PC scores between grazers and dabble/filter feeders (*β* = 0.06, s.e. = 0.03, *z* = 1.88, *p* = 0.06), nor between birds diving into substrate and open water (*β* = −0.06, s.e. = 0.04, *z* = 1.30, *p* = 0.20). Species diving in open water and substrate generally had greater variation in binocular field shape with higher PC1 scores, and thus wider binocular fields (above, below and at the horizontal plane) in comparison to dabbling/filtering species, while the binocular field shape of grazing species was highly conserved (figure 3*b*). Neither log_10_ body mass (*β* = 0.04, s.e. = 0.05, *z* = 0.82, *p* = 0.41) nor primary diet were key predictors for PC1, based on comparison between dietary categories: invertebrate feeders and plant/seed feeders (*β* = 0.88, s.e. = 2.32, *z* = 0.38, *p* = 0.79); omnivores and plant/seed feeders (*β* = −3.67, s.e. = 2.88, *z* = 1.28, *p* = 0.20); invertebrate feeders and omnivores (*β* = −0.02, s.e. = 0.03, *z* = 0.47, *p* = 0.64). For PC2, model selection identified a single top-ranked model (ΔAICc < 2) containing only the predictor of log_10_ body mass (electronic supplementary material, table S7). PC2 increased with log_10_ body mass (*t_37_* = 2.14, *p* = 0.04; electronic supplementary material, figures S3 and S4).

The reconstructed ancestral states of the PC1 and PC2 scores for the phylogeny of Anatidae are illustrated (electronic supplementary material, figures S4 and S5). For foraging behaviour, the PC1 scores were relatively conserved for species that dabble/filter feed or graze, yet PC1 scores were more varied for diving species that forage in substrate. For body mass, the PC2 scores varied widely in relation to log_10_ body mass. While foraging behaviour probably drives variation in PC1 (binocular field shape at and around the horizontal plane), body mass influences interspecific variability in PC2 significantly (binocular field shape associated with the vertical plane), with relatively little correlation in the residual error of the models linked to phylogenetic relationships.

To determine the impact the small dive open category (*n* = 3) was exerting on the findings, we re-ran all analyses without the dive open category included (for full outputs, see the electronic supplementary material, tables S8–S10). These analyses were not conducted for PC2, as the null model was the highest-performing model. For maximal binocular field width, the key findings from the full model (see above) were that dabbling/filtering species were significantly smaller than species diving in open water; removing open water as a category removed this difference (electronic supplementary material, table S8). For angular separation, all findings remained the same, except now there is a significant difference between the dietary categories of omnivores and invertebrate feeders, with omnivores having a higher angular separation (*β* = 31.79, s.e. = 12.33, *z* = 2.58, *p* = 0.01) (electronic supplementary material, table S9). For vertical field extent, in the original models dabbling/filter-feeding species had significantly greater vertical binocular extents than grazing species, and this significant difference persists. No predictors were significant, however, with the null model performing best (electronic supplementary material, table S10). Without the dive open category present, the key findings for PC1 remain the same, with the differences between foraging categories matching that in the original model.

## 4. Discussion

Foraging behaviour, rather than diet, was the primary driver of binocular field size and binocular shape associated with the horizontal plane among the Anatidae, while body mass was the primary driver of the vertical extent of binocular field shape. Phylogenetic relatedness was not a key determinant of binocular field size or shape, although it did exert some influence on the vertical extent of the binocular field. The variation in binocular field characteristics probably reflect the different perceptual challenges experienced by different species when foraging in aquatic and terrestrial environments. Our findings provide strong support for the prediction that binocular field variation among birds is primarily associated with foraging activities rather than shared ancestry [59].

### (a) Foraging behaviour

Foraging behaviour primarily determined three parameters of binocular field size and shape: maximum binocular field width, angular separation and the binocular field shape at and around the horizontal plane (based on PC1 scores). Both foraging behaviour and phylogeny determined vertical binocular field extent, which highlighted the combined influence of foraging technique and species relatedness on this binocular field parameter.

Dabbling and filter-feeding species had small maximum binocular field widths, large angular separation sizes and small binocular field shapes at and around the horizontal plane. These findings probably relate to their reliance on tactile cues from bill-tip organs and bill lamellae for detecting food [12,60,61] since they have less need for accurate bill control. The importance of bill-tip tactile sensitivity in these species is indicated by the very high numbers of tactile units found in their mandible bill-tip organs [12]. This is exemplified by pink-eared ducks *Mal. membranaceus* [44] and mallards *Anas platyrhynchos* [29] that filter feed on planktonic organisms and have maximum binocular field widths of 19.6° and 22.0°, respectively. Dabbling marbled ducks *Marmaronetta angustirostris* have the narrowest maximum binocular field width of 17.3° and they may be less reliant upon visual cues for foraging since they feed close to the water's surface primarily using bill dipping [62].

Dabbling and filter-feeding species had significantly greater vertical binocular extents than grazing species, which reduces the size of the blind area above and below the head. The former foraging group have higher densities of mechanoreceptive formations in their bill-tip organ [12] compared to the latter. This probably increases their tactile sensitivity for the location of food items, hence visual information from the binocular field is probably less important for their feeding. Their reduced reliance upon the binocular field for foraging activities may be traded off against an increased ability to detect predators, when reduction in blind area size provides more visual information from the lateral and posterior fields [10,25]. The dorsally positioned eyes of mallards and northern shovelers [29,46] provide the most extensive binocular field lengths (210° for both) thus enabling comprehensive visual coverage of the celestial hemisphere [59]. During their foraging activities they can gather visual information from all around and above the head, which is important for predator detection [29].

By contrast, diving species that forage from the water column had the widest maximum binocular fields and largest binocular field shapes at and around the horizontal plane. This may reflect the perceptual challenges of chasing and capturing mobile prey from the water column [63], which requires information from the binocular field to achieve accuracy in the direction and timing of bill position to capture evasive prey [25]. For example, smews *Mergellus albellus* (maximum binocular width 33.5°) dive to catch a variety of small fishes [64], while blue ducks *H. malacorhynchos* (maximum binocular field width 34.4°) chase mobile invertebrates, such as mayfly (Ephemeroptera) and stonefly (Plecoptera) larvae [44]. This idea is supported by seabirds from other taxa which dive in open water (e.g. auks and penguins [23]) that also have large maximum binocular field widths. Waterfowl diving to capture prey may require greater maximum binocular field widths (as measured in air) because of the reduction in width that occurs when the eye enters water (owing to loss of corneal refraction), as in penguins and albatrosses [65–67].

Anatidae species diving to obtain food from the substrate had large mean angular separation sizes (the bill tip is furthest from the maximum binocular field width), as they may be less dependent upon visual discrimination of food items in the bill. During winter, sea ducks (Merginae; e.g. buffleheads *Bucephala albeola* and common eiders *Somateria mollissima*) will forage on molluscs and crustaceans taken from the seabed at variable depths [38]. Foraging often takes place in turbid conditions which reduce prey visibility even at close range [81]. Diving white-headed ducks *Oxyura leucocephala* had the greatest angular separation (75°), which probably relates to their winter foraging on saline lakes where they consume submerged aquatic vegetation and invertebrates in brackish water conditions [38], a task for which they probably do not require precise bill positioning. Other avian species for which precision bill control is not required for foraging, such as woodcocks *Scolopax rusticola*, also have their bill projection at the periphery of the binocular field [68] that leads to increased angular separation.

Grazers (the majority being goose and swan species) had small maximum binocular field widths, small angular separation sizes, small vertical binocular extents (this parameter was also influenced by phylogeny), and narrow binocular field shapes at and around the horizontal plane. They had significantly smaller angular separation (the bill tip is nearer to the maximum binocular field width) as compared to species diving in substrate and dabbling or filtering for food. Differences in bill tip position have previously been described for grazing Eurasian wigeons *Mar. penelope* and filter-feeding northern shovelers [29]. Grazing birds may use the smaller angular separation to target specific types of food that are grazed selectively; for example, Canada geese have a small angular separation (−10°) and are able to visually inspect objects at their bill tip to facilitate their selective grazing of highly nutritious vegetation [27]. Other avian species (e.g. herons [34]) also have their bill projections falling approximately centrally within the binocular field (closer to the maximum binocular field width) to enable high precision control of bill position during foraging.

Grazing waterfowl's small vertical binocular extents (hence large blind area sizes) would limit their ability to detect predators in relation to visual field function. However, other aspects of ocular anatomy in grazing species may compensate for this limitation. For example, the presence of an oblique visual streak (high ganglion density) in the retina of Canada geese provides high visual acuity [27]. Thus when their head position has the bill parallel to the ground, they can see the ground and sky simultaneously, which is beneficial for detecting aerial and terrestrial predators [27]. Further research is required to determine whether there are other ocular adaptions in grazing species that may balance the tasks of foraging, predator detection and conspecific observation.

### (b) Body mass

Body mass was the primary predictor of binocular field shape associated with the vertical plane, based on PC2 scores. Anatidae species with higher body mass had greater PC2 scores representing the shape at the upper edge of the binocular field becoming narrower (electronic supplementary material, figure S2). The relationship between body mass and this aspect of binocular field shape may be linked to eye size, since allometric analyses have demonstrated eye size (based on eye diameter) being proportional to body mass in birds [69]. Some high mass avian species with large eyes, including eagles, vultures and hornbills [70] have optical adnexa (e.g. enlarged brows, hair-like feathers on eyelids) that function as sunshade devices to reduce the chance of the sun being imaged on the retina. The presence of sun avoiding enlarged brows, as found in some [30] raptor species, leads to a large blind area over the head that reduces binocular field width at the upper edge. Our study showed that high mass waterfowl (with potentially larger eyes) had narrow binocular field shapes at the upper edge, which leads to questions about potential anatomical adaptions to avoid sun dazzling that may have influenced this aspect of binocular field shape.

Body mass may influence visual parameters in other ways. In general, larger species are less vulnerable to attacks owing to their size [71]. For larger species, a strategy of attempted concealment is less common as size alone can be considered effective enough to deter predation [72]. Previous ideas have posited that if flocking in birds itself evolved owing to predation, flocking should be more prevalent in smaller bird species. In some instances, this idea has held true (e.g. Guianan tropical forest birds [71]). However, other studies [72] have demonstrated either no effect of size on flocking tendencies, or a positive relationship between body mass and the likelihood to form flocks. A positive relationship may indicate a simple aggregation of individuals around a resource rather than being predation driven. In the Anatidae, being in flocks, particularly for swans and geese, may be about shared navigation, learning migration routes and energetic benefits through v-formation flocking [73,74]. Body mass is also related to the distances at which birds are able to detect potential predators; larger bird species have higher detection distances and higher flight initiation distances [75]. Larger birds will, however, travel further to reach cover or safety after fleeing a predator. In Anatidae, this is more prevalent for terrestrial grazing species (swans, geese, wigeons), which return to water for safety [57]. Swaddle & Lockwood [76] found that rather than body mass, it was wing shape and hind limb length that influenced interspecific variation in predation rates. This is believed to be linked to the relationship between body mass and take-off ability; larger birds take longer to take off. Moreover, larger species have comparatively lower energy requirements than those of smaller species [77], and thus it is likely larger species would not tolerate a high-risk scenario, and are generally less tolerant of predator approaches. This is despite larger birds typically being harder to catch and subdue.

## 5. Conclusion

Our study provides to our knowledge, the first, phylogenetically informed, comparative analysis of visual fields in 39 species of ducks, geese and swans, showing that variation in binocular topography and morphology are highly related to foraging behaviour, with limited influence from phylogeny and body mass. We propose that the plasticity of binocular field dimensions and shape can be explained primarily by differences in foraging behaviour traits, however, shared ancestry has some influence on the vertical extent of the binocular field. We recommend using both unidimensional and morphometric approaches in future visual field comparative studies across other avian taxa within a phylogenetically informed framework. These could include a wider variety of relevant life-history traits (e.g. chick type, nest type, prey or predator role) to further address evolutionary questions on avian vision. We also recommend incorporating phylogenomic techniques to better understand the genetic controls on ocular structures that determine binocular vision [78] across avian species.

## Data Availability

The accompanying datasets are available as the electronic supplementary material [79], and at: https://figshare.com/s/faf74ea777228de4fa1d [80].
